# Heightened affective response to perturbation of respiratory but not pain signals in eating, mood, and anxiety disorders

**DOI:** 10.1371/journal.pone.0235346

**Published:** 2020-07-15

**Authors:** Rachel C. Lapidus, Maria Puhl, Rayus Kuplicki, Jennifer L. Stewart, Martin P. Paulus, Jamie L. Rhudy, Justin S. Feinstein, Sahib S. Khalsa, on behalf of the Tulsa 1000 Investigators

**Affiliations:** 1 Laureate Institute for Brain Research, Tulsa, OK, United States of America; 2 Department of Psychology, University of Tulsa, Tulsa, OK, United States of America; 3 Oxley College of Health Sciences, University of Tulsa, Tulsa, OK, United States of America; Ryerson University, CANADA

## Abstract

Several studies have recently suggested that an abnormal processing of respiratory interoceptive and nociceptive (painful) stimuli may contribute to eating disorder (ED) pathophysiology. Mood and anxiety disorders (MA) are also characterized by abnormal respiratory symptoms, and show substantial comorbidity with ED. However, no studies have examined both respiratory and pain processing simultaneously within ED and MA. The present study systematically evaluated responses to perturbations of respiratory and nociceptive signals across the levels of physiology, behavior, and symptom report in a transdiagnostic ED sample (*n* = 51) that was individually matched to MA individuals (*n* = 51) and healthy comparisons (HC; *n* = 51). Participants underwent an inspiratory breath-holding challenge as a probe of respiratory interoception and a cold pressor challenge as a probe of pain processing. We expected both clinical groups to report greater stress and fear in response to respiratory and nociceptive perturbation than HCs, in the absence of differential physiological and behavioral responses. During breath-holding, both the ED and MA groups reported significantly more stress, feelings of suffocation, and suffocation fear than HC, with the ED group reporting the most severe symptoms. Moreover, anxiety sensitivity was related to suffocation fear only in the ED group. The heightened affective responses in the current study occurred in the absence of group differences in behavioral (breath hold duration, cold pressor duration) and physiological (end-tidal carbon dioxide, end-tidal oxygen, heart rate, skin conductance) responses. Against our expectations, there were no group differences in the response to cold pain stimulation. A matched-subgroup analysis focusing on individuals with anorexia nervosa (*n* = 30) produced similar results. These findings underscore the presence of abnormal respiratory interoception in MA and suggest that hyperreactivity to respiratory signals may be a potentially overlooked clinical feature of ED.

## Introduction

Eating disorders (ED) are deadly illnesses that often begin in adolescence or young adulthood, and maintain a chronic course associated with severe impairments of emotional, social, cognitive, and physical functioning, and a low quality of life [1–4]. Despite the devastation caused by these disorders, many of the pathophysiological mechanisms underlying the development and maintenance of ED remain poorly understood.

Interoceptive dysfunction is one component that has been conceptually implicated in ED expression [5]. For example, it has been suggested that the symptoms of certain EDs may be influenced by a perceptual amplification of physiological signals such that changes in the internal body state induce a hyperreactivity characterized by fearful responses to food related stimuli [6]. Supporting this notion are findings that individuals with bulimia nervosa (BN) experience heightened stomach fullness after a standardized non-nutrient water load [7] and individuals with anorexia nervosa (AN) exhibit increased activation of the insular cortex during interoceptive attention to stomach sensations [8]. BN individuals report higher symptoms of anxiety and panic than healthy comparisons (HC) in response to breathing 35% carbon dioxide (CO_2_)-enriched air, but at similar levels as individuals with panic disorder, suggesting that amplifications of interoceptive processing might also extend to respiratory signals [9]. In previous interoceptive processing using the adrenaline analogue isoproterenol, we found that individuals with AN reported a greater intensity of cardiorespiratory sensations relative to HC during meal anticipation, an effect that was particularly pronounced for sensations of dyspnea [10]. These individuals were less accurate at localizing cardiovascular sensations [11], suggesting an inability to accurately discriminate interoceptive signals from different regions in the body despite a tendency to perceive them more intensely.

Nociceptive signals are often considered a component of interoceptive processing [12], and while studies have investigated differences in pain processing in ED, the available evidence is mixed. Acutely ill individuals with AN and BN demonstrate higher pain thresholds to heat stimuli [13–17], and BN show higher pain thresholds to pressure stimuli [15, 16]. However, recovered AN report more unpleasantness during heat pain stimulation than HC in the context of decreased insula responses [18]. These findings suggest that perceptions of heat and pressure pain intensity may be dampened despite affectively experiencing these signals as more aversive. In contrast to studies of heat pain, studies assessing cold pain have not found any evidence of elevated pain thresholds in acute AN or BN patients [19, 20]. Some of these studies were distinguished by methodological limitations (e.g. using suboptimal cold pain stimulation via placing a back of ice on the hand instead of the standard hand immersion in circulating icy water [19]) suggesting that further investigation of cold nociception is warranted.

Over 70% of individuals with EDs exhibit at least one comorbid lifetime mood or anxiety disorder (MA) [21], suggesting that disentangling the interoceptive pathophysiology of ED will require an effort to account for the routinely high rates of comorbidity that are observed with MA. This is especially important as interoceptive dysfunction has also been implicated in the pathophysiology of these disorders [22]. For example, similarly to individuals with EDs, individuals with MA exhibit higher self-reported negative affect and panic symptoms than HC in response to cardiorespiratory physiological challenges, largely in the absence of differential physiological responding [23–25]. There is also mixed evidence for abnormal pain processing in MA disorders [see 26–29]. Therefore, in the current study we planned on the inclusion of a MA control group (as recommended previously [30]), using a standardized set of interoceptive and nociceptive stimulation protocols.

Anxiety sensitivity (AS) [31] is a transdiagnostic trait that may explain abnormal interoception across both ED and MA disorders, based on a tendency towards hyperreactivity to anxiety-inducing cues. For example, when compared to low-AS counterparts, non-clinical individuals with high AS are more likely to report panic symptoms during respiratory challenges [32–34]. Heightened AS is also strongly associated with fearful appraisals of pain, but modestly associated with pain tolerance [35], suggesting the possibility that AS influences the processing of sensory signals beyond those involved in anxiety. AS and responses to respiratory perturbations have been studied extensively in individuals with anxiety disorders (see [36–38] for meta-analytic reviews). However, although existing evidence supports a role for AS in anxiety expression, it has received limited study in ED [39–41].

In the current study we examined physiological, sensory and affective responses to the modulation of two different aversive physiological states (inspiratory breath holding or hand immersion in cold water), in ED and MA participants relative to demographically matched HCs. During both tasks we measured biological reactivity in the form of physiological signal changes (HR, RR, SCL), subjective responses via self-reported intensity and valence of the associated emotional feeling, and objective behavioral responses (exhalation or hand withdrawal). Although interoceptive accuracy is a common experimental measure relating the closeness of a self-report of physiological signal with the actual signal, we did not measure this in the current study as it is not typically measured in these tasks. We selected these probes on the basis that they are minimally invasive, quickly administered, perturb physiological, behavioral and symptom parameters, and can be readily translated to clinical settings. Our goal was to examine whether the acute modulation of respiratory or nociceptive signals elicits abnormal processing in a transdiagnostic sample of ED (spanning the ED diagnoses of AN, BN, or an unspecified eating disorder) in relation to a demographically matched transdiagnostic sample of individuals with mood and/or anxiety disorders (MA) or to demographically matched HCs. We were interested in whether individuals with EDs would demonstrate similar responses to individuals with MA on both tasks, potentially suggesting the possibility of shared underlying etiological processes. Given the inherent heterogeneity of transdiagnostic samples, and the preponderance of studies focusing exclusively on AN, we planned to conduct a sub-group analysis in individuals with this diagnosis to examine whether abnormalities of respiratory and pain processing would be restricted to this category.

For the current study, we tested: 1) whether physiological responses to breath holding and cold pain (indexed via heart rate (HR), electrodermal activity (EDA), respiration rate (RR), or concentrations of CO_2_ or oxygen (O_2_) would differentiate the ED, MA, or HC groups; 2) whether negatively-valenced subjective experiences of breath holding and cold pain (indexed by ratings of stress, suffocation feelings, suffocation fear for breath hold, and pain intensity for cold pressor) would be greater than HC for either the ED group, MA group, or both; and 3) whether affective responses to interoceptive perturbations would relate to anxiety sensitivity within each group.

## Materials and methods

### Participants

Participants were drawn from two studies at the Laureate Institute for Brain Research, one of which included the Tulsa 1000, a naturalistic study focused on assessment and prediction of psychiatric outcomes in 1000 treatment-seeking individuals with MA, substance use, ED, or HC [42]. We examined data from a total of 153 participants: 51 individuals (49 female) with current or lifetime ED who were individually matched using age, sex, gender, and body mass index (BMI) to 51 individuals with current MA, and 51 HC. Individuals with lifetime EDs were allowed to be included in the ED group, as previous findings have suggested that abnormal characteristics associated with ED diagnosis, such as perfectionism, cognitive rigidity, and extreme food preferences can persist after recovery [43–45]. These characteristics in the recovery period are paralleled by maintenance of altered neural responding [46–48], suggesting the residual presence of an ‘illness scar’ that can elucidate pathophysiological processes underlying ED. All participants were required to have a BMI >17 in order to avoid acute physiological-related confounds due to severe malnutrition [49].

Participants were diagnostically grouped based on a structured diagnostic clinical interview (MINI 6.0 or 7.0) [50]. To be included in the ED group, participants were required to meet current/lifetime diagnostic criteria for AN, BN, or an eating disorder not otherwise specified. ED participants were permitted to have co-occurring MA disorders given the high comorbidity rates of these disorders [51, 52]. Inclusion in the MA group required the current diagnosis of a mood or anxiety disorder via the MINI interview, and a self-rated screening scale score of ≥ 10 on the Patient Health Questionnaire-9 (PHQ-9) [53] and/or ≥ 8 on the Overall Anxiety Severity and Impairment Scale (OASIS) [54]. A score of < 2 on the Sick, Control, One, Fat, Food (SCOFF) Questionnaire [55] and/or absence of a current or prior history of an ED, as determined through a structured life chart interview [56] was also required to be in the MA group. Individuals in the HC group were negative for any psychiatric disorder per the MINI interview, and reported symptom scores beneath the clinical cutoffs on all of the self-rated screening scales. Psychosis, bipolar disorder, moderate-to-severe traumatic brain injury or other neurocognitive disorder, and pregnancy were exclusionary for all participants. Additionally, individuals reporting active suicidal ideation with intent or plan were excluded for safety reasons and were provided with appropriate clinical resources. Obsessive compulsive disorder was exclusionary for the Tulsa 1000 study. Patients taking psychotropic medications were allowed, provided the dose had been stable for at least two weeks. Written informed consent was obtained for all participants as approved by the Western Institutional Review Board screening protocol #20101611, and all subjects received compensation for their participation.

### Procedure

Participants were asked to abstain from central nervous system stimulants and depressants such as caffeine, nicotine, amphetamines, or alcohol the day of their appointment. Participants were instructed by computer as they completed a series of behavioral tasks, including the inspiratory breath hold and cold pressor tasks. The breath hold and cold pressor tasks were separated by a heartbeat tapping task that was not reported on in this study. In the heartbeat tapping task, individuals were asked to focus on the sensation of their heartbeat and to press a key on a keyboard when they felt their heartbeat. During this trial, participants were also asked to hold their breath while tapping to amplify interoceptive sensations. This task lasted for a maximum of one minute followed by approximately 30 seconds of time in which participants entered subjective ratings of the task using a keyboard. The cold pressor task began next, with approximately three minutes of instruction and equipment set-up occurring prior to the participant submerging their hand in the water. Altogether, this resulted in a three-and-a-half minute delay between the final breath hold in the heartbeat tapping task and the cold pressor task, allowing ample time for recovery following a breath hold in the previous task [57]. For a minority of participants, the breath hold and cold pressor tasks were conducted on different days due to technical difficulties. In these instances, participants were rescheduled to complete the cold pressor task at their earliest convenience (typically less than a week later).

Physiological signals measured during these tasks were collected continuously at 1000 Hz and included the electrocardiogram (ECG; lead II configuration), electrodermal activity (electrodes placed on the middle phalanx of index and middle fingers), and respiration (via transducer belt), (MP510, Biopac Systems Inc., Lehigh, Pennsylvania). Percent oxygen (O_2_) and carbon dioxide (CO_2_) concentrations were collected prior to and following breath hold trials using an Oxigraf capnometer (Sunnyvale, CA).

#### Inspiratory breath hold (BH) challenge

The BH challenge consisted of two trials (BH1 and BH2), each limited to a two-minute maximum duration breath hold [58]. Two trials were used as previous work has shown increased breath hold times following repeated trials [59]. To avoid biased responding, participants were not informed of the maximum trial duration. Participants were provided with a nose clip to prevent inadvertent respirations. They were then instructed to exhale all the air out of their lungs, take a deep breath in, hold it for as long as they could tolerate, and then exhale into the capnometer. BH trials were separated by approximately a two-minute recovery period. Immediately afterwards participants rated the subjective effort, unpleasantness, intensity, difficulty, stress, breathlessness, urge to breathe, feelings of suffocation, and suffocation fear experienced during the task using a visual analogue scale (VAS) that ranged from 0 (“not at all”) to 100 (“extremely”).

#### Cold pressor (CP) challenge

The CP challenge consisted of one trial with a two-minute maximum duration. Participants were instructed to keep their dominant hand submerged up to the wrist in cold water continuously circulating at a constant temperature of five degrees Celsius (Thermo Fisher Scientific, Pittsburgh, PA) for as long as they could tolerate. To avoid biased responding, participants were not informed of the maximum trial duration. Throughout the challenge, participants made continuous real-time ratings of their pain intensity as it increased and decreased on a scale ranging from 0 (no pain) to 100 (worst pain imaginable). These ratings were used to calculate each individual’s peak pain rating, as well as the time to reach mild (25/100), moderate (50/100), and peak pain. Immediately afterwards they rated the degree of unpleasantness, stress, and difficulty experienced on a visual analogue scale (VAS) ranging from 0 (none) to 100 (extremely).

#### Clinical symptoms

Self-report scales were used to quantify ED pathology including the Eating Disorder Diagnostic Scale (EDDS) [60] and the Eating Disorder Examination Questionnaire-6 (EDE-Q) [61]. All participants completed the Anxiety Sensitivity Index-3 (ASI), a transdiagnostic trait measure of the fear of experiencing anxiety-related symptoms and sensations [31].

### Physiological data processing

Physiological data were imported into R version 5.3.1 (R core team, 2013) for analysis. Outliers (e.g., signal interference or movement associated artifacts) were identified through a manual quality-control process and corrected by using a two-sided moving average linear filter. To determine how the breath hold and cold pressor tasks affected physiology, changes in heart rate, skin conductance, O_2_, and CO_2_ were calculated. For the heart rate, maximum increase/decrease change scores were calculated by subtracting the baseline heart rate (defined as the average heart rate during the 20 seconds prior to trial onset) from the maximum and minimum observed heart rate using a five second moving average window. A similar calculation was done with skin conductance. Average O_2_ and CO_2_ concentrations were examined before and immediately after each BH trial, and O_2_ and CO_2_ change scores were calculated by subtracting post-trial from pre-trial concentrations. Average heart rate and electrodermal activity (via skin conductance level, SCL) was also calculated across the entire window within each BH trial, as well as across the CP task.

### Statistical analysis

To examine differences in BH responding, a mixed analysis of variance (ANOVA) was computed with group (ED, MA, and HC) as the between-subjects factor and trial (BH1, BH2) as the within-subjects factor for the following dependent variables: average HR, maximum HR increase, maximum HR decrease, average SCL, maximum SCL increase, maximum SCL decrease, pre/post CO_2_ change, pre/post O_2_ change, BH duration, and VAS ratings for effort, unpleasantness, intensity, difficulty, stress, urge to breathe, feelings of suffocation, and suffocation fear. For CP responses, one-way ANOVAs were conducted with group as the between-subjects factor for each of the following dependent variables: average HR, maximum HR increase, maximum HR decrease, average SCL, maximum SCL increase, maximum SCL decrease, trial duration, time to mild pain, time to moderate pain, time to peak pain, peak pain, and VAS ratings for unpleasantness, difficulty, and stress. Standard listwise deletion was used in the analysis to address missing data. Missing values were not imputed due to the large number of variables relative to the number of participants in each group. All results were corrected for multiple-comparisons using the Benjamini-Hochberg procedure. Significant ANOVA group main effects and interactions were evaluated via post-hoc two-sided t-tests. As no group by trial interactions were identified on the breath hold trials 1 and 2, average scores were calculated for use in t-tests. In the subgroup analysis, the same tests were conducted using only AN (*n* = 30) and individually AN-matched MA participants (*n* = 30) and HC (*n* = 30) participants. The small BN sample size (*n* = 14) prevented a comparable subgroup analysis. All analyses were conducted using R version 5.3.1 (R core team, 2013).

## Results

### Participant characteristics

The groups did not differ in age, sex, or BMI, but they differed with respect to measures of psychopathology (Table 1). The ED group endorsed significantly higher SCOFF scores than the MA group, who in turn endorsed significantly higher scores than HC. The ED and MA groups reported significantly higher OASIS, PHQ-9, ASI total, ASI cognitive concern, ASI physical concern, and ASI social concern scores than HC, but these groups did not differ from each other. The ED and MA groups exhibited generally similar amounts of comorbid disorders (Table 1), while the ED group had a significantly higher percentage of medicated individuals than the MA group (Table 1). Average ED severity scores were above the minimum cutoffs for both the EDDS and EDE-Q, indicative of a clinical ED sample (EDDS: average score = 33.9, 16.5 cutoff, [62]; EDE-Q: average score = 2.68, 2.30 cutoff [63]).

**Table 1:** Demographics and screening scores at study entry.

	Mean (SD)			*F*	*df*	*p*
	HC	MA	ED			
Sample Size	n = 51 (2 male)	n = 51 (2 male)	n = 51 (2 male)			
**Demographics**						
Age, years	26.57 (7.63)	28.61 (9.06)	25.82 (7.27)	1.65	2, 150	0.20
BMI	23.05 (3.36)	22.37 (2.93)	22.39 (3.44)	0.71	2, 150	0.49
Psychotropic medications	0%	33%	65%	χ^2^ = 10.04	2, n = 102	**<0.01**
**Clinical Measures**						
SCOFF^a^	0.10 (0.36)	0.76 (1.01)	3.11 (1.53)	110.0	2, 150	**<0.0001**
OASIS^b^	0.94 (1.32)	9.33 (3.15)	10.06 (4.12)	137.5	2, 150	**<0.0001**
PHQ-9^b^	0.67 (1.19)	12.00 (4.54)	12.04 (6.51)	102.0	2, 150	**<0.0001**
ASI-Total^b^	6.84 (5.01)	26.04 (15.11)	29.12 (14.14)	49.05	2,149	**<0.0001**
ASI—Physical^b^	1.25 (1.57)	6.27 (5.44)	6.36 (5.64)	20.47	2, 149	**<0.0001**
ASI–Cognitive^b^	0.67 (1.35)	6.86 (6.95)	9.04 (6.07)	33.04	2, 149	**<0.0001**
ASI–Social^b^	4.92 (3.40)	12.90 (6.28)	13.72 (5.58)	43.81	2, 149	**<0.0001**
Anorexia Nervosa	-	-	30 (58.9%)	-	-	-
Bulimia Nervosa	-	-	14 (27.5%)	-	-	-
Unspecified Eating Disorder	-	-	7 (13.7%)	-	-	-
Major Depressive Disorder—LT	-	47 (92.2%)	49 (96.0%)	-	-	-
Generalized Anxiety Disorder	-	21 (41.2%)	34 (66.7%)	-	-	-
Social Phobia	-	16 (31.4%)	13 (25.5%)	-	-	-
Panic Disorder	-	8 (15.7%)	6 (11.8%)	-	-	-
Agoraphobia	-	5 (9.8%)	2 (0.40%)	-	-	-
Post-Traumatic Stress Disorder	-	14 (27.5%)	10 (19.6%)	-	-	-
Alcohol/Substance Use Disorder—LT	-	5 (9.8%)	9 (17.6%)	-	-	-
Obsessive Compulsive Disorder	-	0 (0.0%)	1 (0.02%)	-	-	-

HC = Healthy Comparison, MA = Mood/Anxiety, ED = Eating Disorder, BMI = Body Mass Index, Meds = Medication, SCOFF = Sick, Control, One Stone, Fat, Food Eating Disorder Screener, OASIS = Overall Anxiety Severity and Impairment Scale, PHQ-9 = Patient Health Questionnaire, ASI = Anxiety Sensitivity Index. LT = Lifetime

^a^ED>MA>HC

^b^ED and MA > HC. Bolded values indicate *p* < 0.05

### Missing data

There was data missing for several of the measures used within our sample. The two primary reasons related to differences in the ED symptom severity scales employed in the two studies, and to equipment malfunction in a subset of cases (e.g. capnometer not accurately recording O2 and CO2 levels during a BH trial). Regarding ED symptom severity self-report measures, 17% of data were missing for the EDDS and 69% of data were missing for the EDE-Q6. ED symptom severity was not used as an outcome measure of this study. For the BH task, less than 1% of the self-report data were missing across each variable and approximately 3–4% of the behavioral data were missing across each variable. For the physiological data in the breath hold task, respiration rate data were missing for between 6–7% for each variable, skin conductance level data were missing for between 2–4% for each variable, heartrate data was missing for between 6%-7% for each variable, with the exception of “heartrate change from baseline to minimum heartrate” for BH1 and BH2, which was missing at 18% and 19% respectively. For both BH1 and BH2, data for O_2_ and CO_2_ levels were missing at rates of 3–4% of each variable, with the exception of the following: baseline O_2_ and CO_2_ (16–21% missing per variable), post O_2_ and CO_2_ (10% missing per variable), and change in O_2_ and CO_2_ from baseline to post breath hold for BH1 and BH2 (19–23% per variable). For the CP task, less than 1% of data were missing across each self-report level variable and between 3–5% of data were missing across each behavioral level variable, with the exception of time to moderate pain, which was missing at 12%. At the physiological level, respiration rate and skin conductance level variables were missing at 3% each, and heartrate data was missing at 8–9% for each variable, with the exception of the variable heartrate change from baseline to minimum heartrate which was missing at 30%. Less than 1% of data were missing for each of the ASI-3 subscales. See S3 File for a detailed description of missing data for each variable sorted by participant group.

### Physiology and behavior

**Fig 1:**
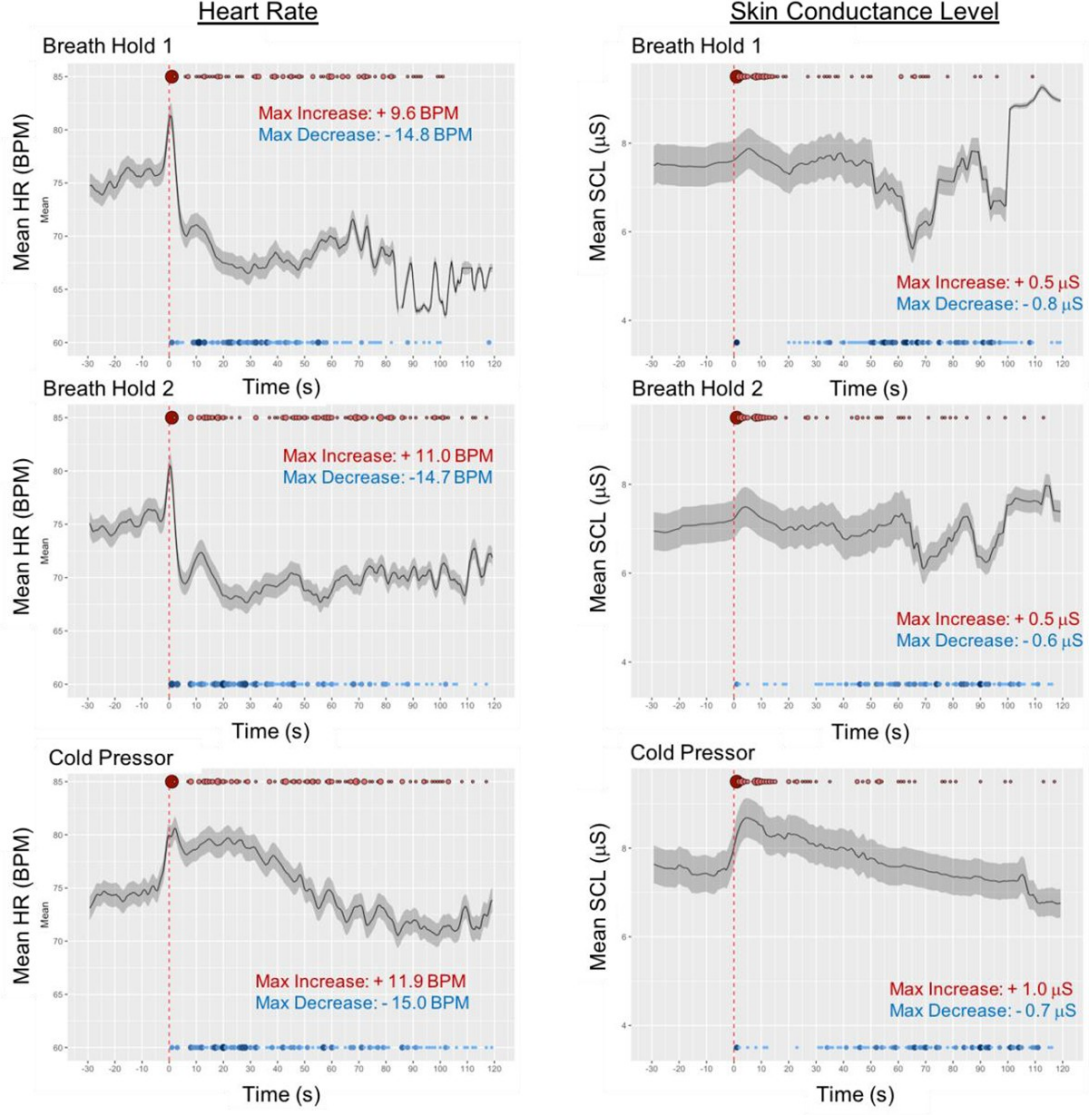
Time course of average Heart Rate (HR) and Skin Conductance Level (SCL) for breath hold and cold pressor. Black line = mean. Grey shaded area = SE. *** indicates significant difference at *p* < 0.001. Time 0 (s) = start of trial (inhale for BH, hand enters water for CP). Mean heart rate (beats per minute, BPM) or skin conductance level (microsiemens, μS) with shaded standard error for the entire sample (n = 144) for each task. Red dashed line indicates task onset. Late fluctuations may be related to subject dropout, as such BH1 was only graphed for 120 seconds. Red circles indicate time at which peak heart rate or SCL was achieved for each individual. Blue circles indicate time at which minimum heart rate or SCL was reached for each individual. Color saturation of circles indicates greater frequency. Individual groups were not plotted because no significant differences were observed between groups (see Table 2).

**Fig 2:**
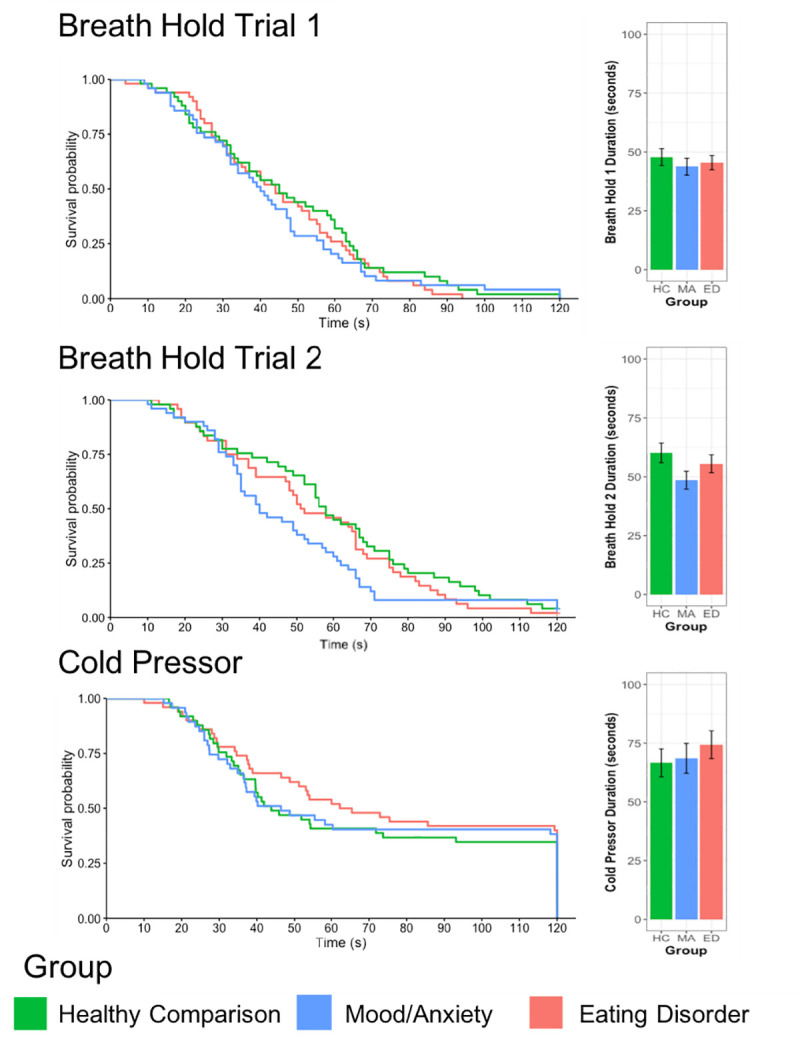
Survival curves for behavioral responses to the breath hold and cold pressor tasks. Exhalation ended the breath hold task. Hand removal from water ended the cold pressor task.

Analysis of the available data revealed that both the BH and the CP tasks successfully modulated autonomic physiology (Fig 1) and behavior (Fig 2), with the majority of participants terminating each task before the maximum trial duration. Across all groups, both the BH and CP probes elicited initial elevations in heart rate followed by a slowing and reduction below baseline levels. However, despite the observed task-induced changes in physiology and behavior, there were no significant group main effects or interactions for either BH or CP (Table 2).

**Table 2:** Between group differences for healthy, mood/anxiety, and eating disorder groups on the breath hold and cold pressor tasks.

Level	Task	Source	*F*	*DF*	*p*	*p*_*corr*_
Physiological	Breath Hold	Avg HR	0.20	2, 140	0.82	0.91
		Maximum HR Increase	0.30	2, 140	0.74	0.91
		Maximum HR Decrease	1.87	2, 130	0.16	0.68
		Avg SCL	0.87	2, 144	0.42	0.91
		Maximum SCL Increase	0.24	2, 144	0.78	0.91
		Maximum SCL Decrease	0.10	2, 144	0.91	0.91
		O^2^ Change	0.70	2, 126	0.50	0.91
		CO^2^ Change	1.83	2, 126	0.17	0.68
	Cold Pressor	Avg HR	0.74	2, 137	0.48	0.58
		Maximum HR Increase	0.44	2, 137	0.65	0.65
		Maximum HR Decrease	1.24	2, 103	0.29	0.58
		Avg SCL	1.19	2, 145	0.31	0.58
		Maximum SCL Increase	1.08	2, 145	0.34	0.58
		Maximum SCL Decrease	0.86	2, 145	0.43	0.58
Behavioral	Breath Hold	Duration	1.20	2, 144	0.30	-
	Cold Pressor	Duration	0.45	2, 143	0.64	0.64
		Time to Mild Pain	0.84	2, 142	0.16	0.21
		Time to Moderate Pain	1.83	2, 131	0.16	0.21
		Time to Peak Pain	2.60	2, 145	0.08	0.21
Symptom	Breath Hold	Effort	0.21	2, 149	0.81	0.81
		Unpleasantness	1.17	2, 149	0.31	0.36
		Intensity	1.15	2, 149	0.32	0.36
		Difficulty	3.29	2, 149	0.04	0.07
		**Stress**	6.34	2, 149	0.002	**0.01**
		Breathlessness	2.12	2, 149	0.12	0.18
		Urge to Breathe	3.24	2, 149	0.04	0.07
		**Feelings of Suffocation**	7.51	2, 149	0.001	**0.01**
		**Fear of Suffocation**	8.86	2, 149	0.0002	**0.002**
	Cold Pressor	Peak Pain	0.08	2, 145	0.92	0.92
		Unpleasantness	2.46	2, 149	0.09	0.27
		Difficulty	1.64	2, 149	0.20	0.27
		Stress	0.74	2, 149	0.17	0.27

*p* = *p* value uncorrected. *p*_*corr*_ = *p* value with Benjamini-Hochberg False Discovery Rate Correction applied. Bolded values indicate significance of *p* < 0.05. No correction was necessary for the solitary Breath Hold Duration test. HR = Heart Rate. SCL = Skin Conductance Level. Maximum HR/SCL Increase indicates the average increase in BPM from baseline to peak heart rate or average increase in μS from baseline to peak SCL. Maximum HR or SCL Decrease indicates the average decrease in BPM orμS from baseline to minimum heart rate.

### Symptom report

**Fig 3:**
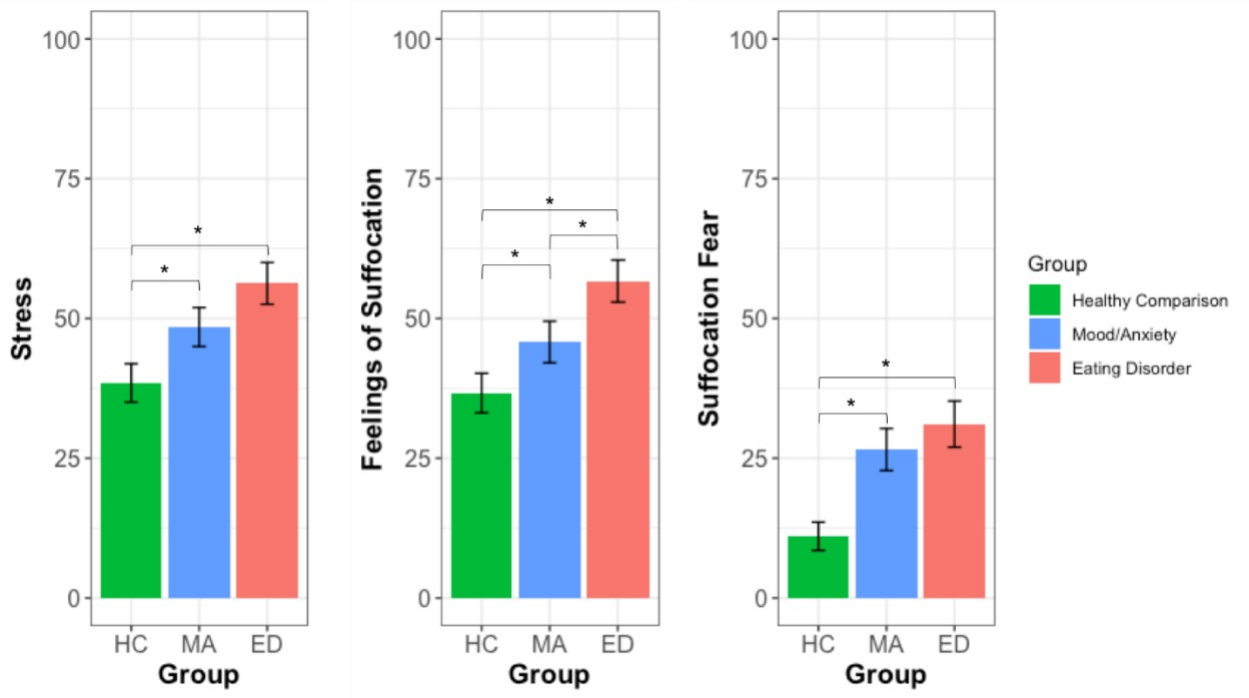
Average feelings of suffocation, stress, and suffocation fear. Ratings could range from 0 ‘not at all’ to 100 ‘extremely’. HC = Healthy Comparison. MA = Mood/Anxiety. ED = Eating Disorder. * indicates significant difference at *p* < 0.05.

Although no between group differences in physiology and behavior were detected on the BH task, there were significant between group differences for reports of stress, *F*(2,149) = 6.34, *p* = 0.01, feelings of suffocation, *F*(2,149) = 7.51, *p* = 0.01, and suffocation fear, *F*(2,149) = 8.86, *p* = 0.002 (Table 2, Fig 3). Reports of effort, unpleasantness, intensity, difficulty, and breathlessness were not significantly different between groups. The MA and the ED groups both reported higher stress and higher suffocation fear than HC in response to breath holding, but they were not significantly different from each other (Table 3). The ED group reported the highest feelings of suffocation, at levels that were significantly greater than both MA and HC, while the MA group reported higher feelings of suffocation than the HC group (Table 3). The ED group showed larger effect sizes than MA for stress (0.69 vs 0.41 Cohen’s *d*) and suffocation fear (0.82 vs 0.68 Cohen’s *d*), although both groups did not significantly differ from each other on these ratings. Finally, despite a clear elicitation of pain ratings within each group, there were no significant between group differences in any of the reported pain symptoms during the CP task (Table 2).

**Table 3:** Follow-up tests for symptom level variables to discern between group differences.

Task	Source	*T*	DF	*p*	*d* (95% CI)
Breath Hold	**Stress: HC vs. MA**	-2.04	99	**0.04**	**0.41** (0.01–0.80)
	**Stress: HC vs. ED**	-3.51	99	**0.001**	**0.69** (0.28–1.10)
	Stress: MA vs. ED	-1.53	99	0.13	0.30 (-0.09–0.69)
	Feelings of Suffocation: HC vs. MA	-1.78	99	0.08	0.35 (-0.05–0.74)
	**Feelings of Suffocation: HC vs. ED**	-3.88	100	**0.0002**	**0.77** (0.35–1.18)
	**Feelings of Suffocation: MA vs. ED**	-2.06	99	**0.04**	**0.41** (0.01–0.80)
	**Suffocation Fear: HC vs. MA**	-3.42	86	**0.001**	**0.68** (0.27–1.10)
	**Suffocation Fear: HC vs. ED**	-4.12	83	**0.0001**	**0.82** (0.40–1.24)
	Suffocation Fear: MA vs. ED	-0.81	98	0.42	0.16 (0.02–0.81)

HC = Healthy Comparison, MA = Mood/Anxiety. ED = Eating Disorder. *d* = Cohen’s *d* effect size. 95% CI = 95% confidence interval for effect size. Bolded values indicate significance of *p* < 0.05. Symptom ratings for each individual were averaged over Breath Hold Trials 1 and 2. See Fig 3 for visual representation.

### AN subgroup analysis

There were no between group differences in age, *F*(2,87) = 0.38, *p* = 0.69, or BMI, *F*(2,87) = 0.60, *p* = 0.55 (S1 Table). Both the AN and MA groups endorsed significantly higher OASIS, PHQ-9, and ASI total/subscale scores than HC but these did not differ from each other. The AN exhibited the highest SCOFF scores, followed by MA and then HC. Similar to the pattern of findings in the larger transdiagnostic sample, there were no group differences in physiology or behavior for BH, and no group differences in any of the CP variables (Table 4). At the symptom level, we observed group differences in feelings of suffocation, *F*(2,87) = 6.16, *p* = 0.027, and suffocation fear *F*(2,87) = 4.88, *p* = 0.045, (Table 4, S1 Fig). Within these, both the AN and MA groups reported higher feelings of suffocation and suffocation fear than HC, but they were not different from each other (S2 Table, S1 Fig). Again, although the AN and the MA groups did not significantly differ, the AN group demonstrated larger effect sizes for both feelings of suffocation (0.91 vs 0.56 Cohen’s *d*) and suffocation fear (0.80 vs 0.60 Cohen’s *d*).

**Table 4:** Between group differences for the anorexia nervosa subgroup on the breath hold and cold pressor tasks.

Level	Task	Source	*F*	DF	*p*	*p*_*corr*_
Physiological	Breath Hold	Avg HR	0.36	2, 86	0.70	0.95
		Maximum HR Increase	0.00	2, 83	1.00	1.00
		Maximum HR Decrease	0.61	2, 78	0.55	0.95
		Avg SCL	1.39	2, 86	0.25	0.95
		Maximum SCL Increase	0.18	2, 86	0.83	0.95
		Maximum SCL Decrease	0.27	2, 86	0.77	0.95
		O^2^ Change	0.34	2, 75	0.72	0.95
		CO^2^ Change	1.99	2, 75	0.14	0.95
	Cold Pressor	Avg HR	0.83	2, 82	0.44	0.53
		Maximum HR Increase	0.86	2, 82	0.43	0.53
		Maximum HR Decrease	0.89	2, 64	0.41	0.53
		Avg SCL	1.71	2, 87	0.19	0.53
		Maximum SCL Increase	1.41	2, 87	0.25	0.53
		Maximum SCL Decrease	0.38	2,87	0.69	0.69
Behavioral	Breath Hold	Duration	0.12	2, 86	0.88	-
	Cold Pressor	Duration	0.37	2, 83	0.69	0.69
		Time to Mild Pain	0.86	2, 84	0.42	0.56
		Time to Moderate Pain	2.35	2, 79	0.10	0.32
		Time to Peak Pain	1.85	2, 85	0.16	0.32
Symptom	Breath Hold	Effort	1.79	2, 87	0.17	0.17
		Unpleasantness	2.05	2, 87	0.14	0.16
		Intensity	3.07	2, 87	0.05	0.09
		Difficulty	2.91	2, 87	0.06	0.09
		Stress	3.88	2, 87	0.02	0.06
		Breathlessness	2.24	2, 87	0.11	0.14
		Urge to Breathe	3.63	2, 87	0.03	0.07
		**Feelings of Suffocation**	6.16	2, 87	0.003	**0.027**
		**Fear of Suffocation**	4.88	2, 87	0.01	**0.045**
	Cold Pressor	Peak Pain	0.30	2, 86	0.74	0.74
		Unpleasantness	0.98	2, 87	0.38	0.51
		Difficulty	1.27	2, 87	0.29	0.51
		Stress	1.17	2, 87	0.31	0.51

*p* = *p* value uncorrected. *p*_*corr*_ = *p* value with Benjamini-Hochberg False Discovery Rate Correction applied. Bolded values indicate significance of *p* < 0.05. BH = Breath hold. CP = Cold pressor. HR = Heart Rate. SCL = Skin Conductance Level. Maximum HR/SCL Increase indicates the average increase in BPM/μS from baseline to peak heartrate. Maximum HR/SCL Decrease indicates the average decrease in BPM/μS from baseline to minimum heartrate.

### Correlational analysis

**Fig 4:**
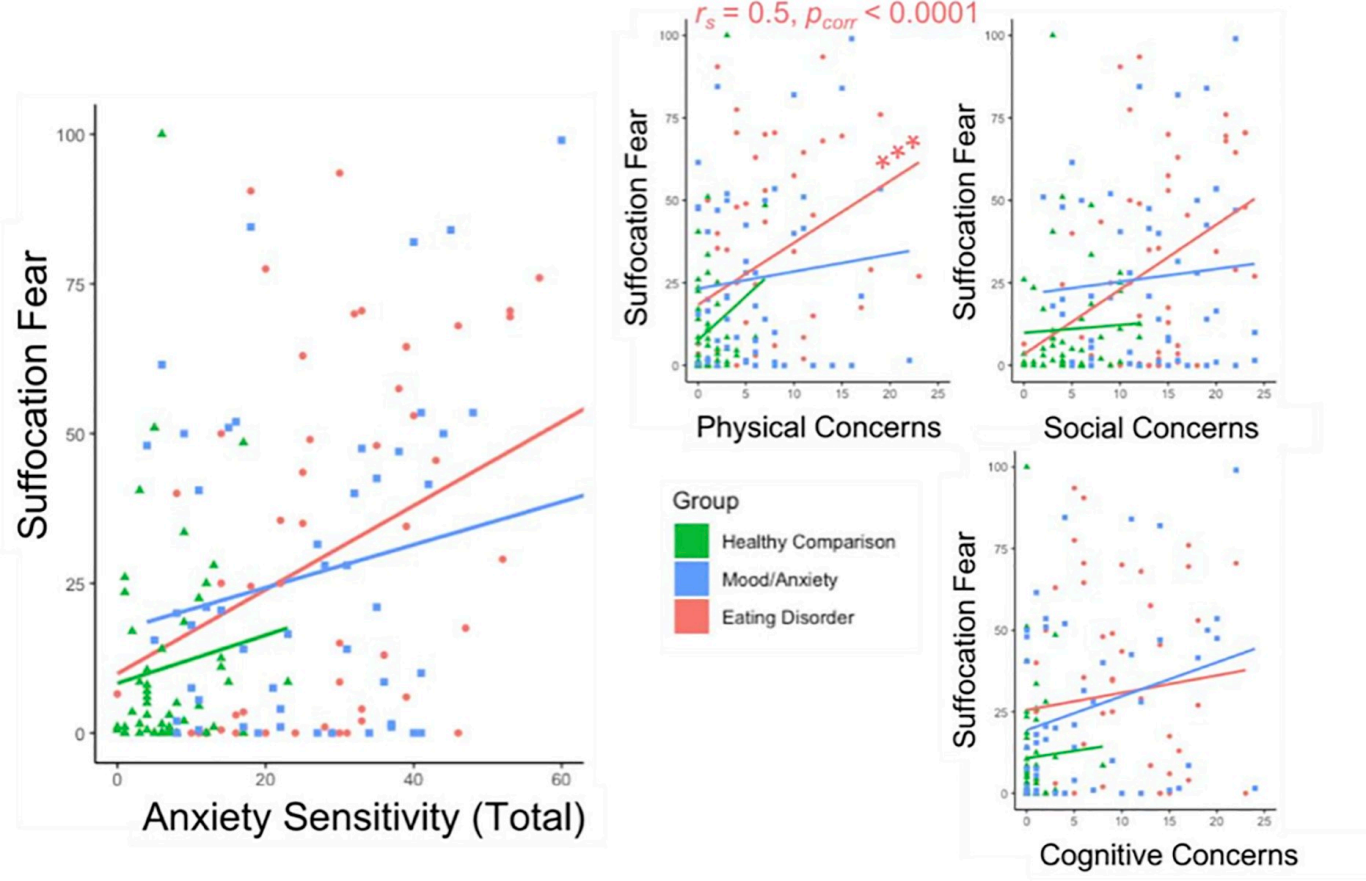
Relationships between anxiety sensitivity and suffocation fear. Suffocation fear ratings could range from 0 ‘not at all’ to 100 ‘extremely’. Insets depict relationships between suffocation fear and anxiety sensitivity index subscales. HC = Healthy Comparison. MA = Mood/Anxiety. ED = Eating Disorder. *p*_*corr*_ = p value with Benjamini-Hochberg correction applied. *** indicates significance at *p* < 0.001.

To examine whether anxiety sensitivity was differentially related to suffocation fear in each group, we calculated Spearman’s rank-order correlations between ASI Total Score and each ASI subscale (cognitive concerns, physical concerns, social concerns) and average suffocation fear (S3 Table). After applying the Benjamini-Hochberg correction for false discovery rates, this analysis revealed that suffocation fears were positively correlated with ASI scores, but only in the ED group for the physical concerns subscale (*r*_*s*_ = 0.50, *p*<0.0001; Fig 4).

## Discussion

The present study examined physiological, behavioral, and subjective responses to the acute modulation of respiratory interoceptive and cold pain signals across demographically matched samples of participants with ED or MA, relative to HC. We observed significant task-related changes in physiological and behavioral responses during both tasks, but without group-level differences in these parameters. However, during inspiratory breath holding, we observed heightened levels of stress, feelings of suffocation, and suffocation fear in both ED and MA relative to healthy individuals, with the ED group reporting significantly higher feelings of suffocation than the MA group after correcting for multiple comparisons. The AN-only subgroup analysis revealed findings similar to the larger transdiagnostic ED sample, suggesting the possibility that heightened suffocation fear may be common across EDs. We did not identify any evidence of abnormal nociceptive responding to cold pain, at any level, for MA, ED, or the AN-only subgroup. Finally, we found that suffocation fears were correlated with anxiety sensitivity physical concerns, but only in the ED group.

Previous studies examining responses to homeostatic perturbations have shown that physiological responses do not differ between ED individuals and HC [10, 64–67], similar to the observations of the current study. While it would be premature to assume that there are no differences in peripheral (autonomic) physiology between these groups, the presence of group differences in symptom reports in the absence of differences in any of the physiological measures recorded in the current study suggests that affective response to breath holding may be localized to neural circuits within the central nervous system responsible for processing interoceptive threat. Confirming this possibility would require subsequent studies employing measurement of neural signals via functional neuroimaging or electroencephalography methods. Alternative processes such as attentional biases, or temperamental traits (e.g. interoceptive sensibility) could also play a role, although we would expect these processes to also be driven by central nervous system mechanisms. Supporting evidence for these arguments comes from prior studies comparing women recovered from AN to HC that observed aberrant anterior and dorsal-mid insular activity during interoceptive attention [8], and increased activation in the striatum, cingulate, and prefrontal cortices during loaded breathing [68] in the absence of clear differences in physiological responses. These findings also suggest some possible cortical loci of abnormal respiratory interoceptive sensation (i.e., insula, striatum, cingulate, frontal cortices). Taken together, these findings and the data from the current study emphasize two points: 1) the importance of measuring responses to physiological challenges across various levels of the nervous system to localize dysfunction, and 2) concomitant measurement of the associated central neural circuits may be necessary to adequately identify anatomical substrates responsible for generating abnormal interoceptive symptom reports.

The observation of heightened suffocation feelings and fear across both ED and MA in the absence of group differences in behavioral or physiological responses to breath holding is evocative of Klein's suffocation false alarm hypothesis [69]. This hypothesis proposed that a hypersensitivity to fluctuations in CO_2_ levels preferentially triggers misperceptions of suffocation in individuals with panic disorder. In congruence with this notion, previous studies have observed heightened anxiety and panic symptoms in various anxiety disorders in response to voluntary breath holding [58] and the 35% CO_2_ inhalation challenge [23, 24, 70]. The fact that a voluntary breath hold could elicit a fear of suffocation seems paradoxical, as subjects are always free to exhale and continue breathing. Yet, the repeated observation of such fears in breath hold studies speaks to the robustness of the phenomena. Our observation in the current study that CO_2_ changes and suffocation fear were not significantly correlated following the breath hold provides intriguing evidence for an argument that the task may have elicited a *false* suffocation alarm in both patient groups. To this point, in the current study the ED group actually demonstrated the lowest changes in CO_2_ from pre to post breath hold (though not significantly different), despite reporting the highest fears and feelings of suffocation (S4 Table). These suffocation fears also occurred despite the ED group’s higher rate of psychotropic medication usage, something that has been reported to reduce behavioral responses to CO_2_ exposure [71]. Although preliminary until replicated, we believe that the current findings could be interpreted as evidence in support of a hypersensitivity to CO_2_ in ED. Importantly, these findings suggest that suffocation feelings and fears are not exclusive to anxiety disorders, but are relevant to ED as well.

While this is the first study to explicitly examine suffocation fear in transdiagnostic EDs as well as in AN, previous studies documenting abnormal respiratory processing in ED can be interpreted as providing evidence of suffocation alarm misfires. For example, there are heightened reports of choking following loaded breathing in AN as compared to HC [68], elevated panic attack rates in BN similar to individuals with panic disorder after breathing 35% CO_2_ enriched air [9], and heightened ratings of dyspnea intensity in AN vs. HC during administration of isoproterenol, an adrenaline analogue [10]. In contrast, Perna et al., 2004 [72] found that in response to 35% CO_2_ inhalation, individuals with transdiagnostic EDs responded more similarly to HCs than to panic disorder patients with respect to panic-related sensations (e.g. trembling, palpitations) and subjective ratings. However, this study excluded ED individuals with comorbid diagnoses, and had a substantially smaller sample size than the current study (*n* = 15 per group), which may have contributed to the differences in findings. With this consideration in mind, our study points to interoceptive abnormalities in EDs that are not specific only to the gastrointestinal system, and thus have the potential to expand research and clinical attention to the examination of respiratory processes in ED patients.

Another aim of the current study was to examine whether individuals with EDs display exaggerated reactions to negatively-valenced cutaneous stimuli beyond the respiratory interoceptive domain (i.e., cold pain). In the current study we did not find any evidence of differential responding to cold pain. The lack of elevated self-report ratings during cold pain elicitation indicates that the heightened reports of stress and suffocation in ED cannot be attributable to a general amplification of distress across all domains of aversive sensory processing. Simultaneously, the lack of group differences with cold pain in the context of other studies finding elevated pain thresholds [13, 17] and heightened unpleasant experience in ED [18] to pain modalities (i.e. heat and pressure), suggests there is a complex relationship between nociceptive processing in ED. Perhaps, as in depression [27], the experience of pain is modality specific (e.g., heat pain eliciting different response from cold pain). Although speculative, responses to cold pain stimulation may also be blunted by chronic peripheral vasoconstriction in ED, an allostatic adjustment which is thought to occur in an effort to conserve heat [73]. In other words, we cannot rule out the possibility of a different outcome had we tested cold pain processing in the body trunk (e.g., via whole body cold plunge). Alternatively, as illness acuity influences nociceptive responding to heat pain and remits with unrestrained eating [13, 74], thermal pain thresholds may be a more relevant state factor for understanding pain processing in ED. Our study also cannot offer conclusions about nociceptive processing across each illness phase, as we did not focus our efforts on acutely ill ED individuals. However, this study represents the largest sample to date of cold pain processing in ED, and provides further evidence (i.e., beyond [19, 20]) that abnormal response to cold pain is not a characteristic of EDs and specifically, AN.

Lastly, we sought to examine the relationship between anxiety sensitivity and affective responses to interoceptive and nociceptive perturbations in ED and MA. While anxiety sensitivity is already identified as a transdiagnostic predictor of symptom response during interoceptive and nociceptive challenges in both HC [75] and MA [76, 77], little research has used behavioral measures to examine the relevance of anxiety sensitivity to ED despite the high comorbidity between ED and MA. Our observation that suffocation fears were significantly related to anxiety sensitivity only in the ED group reinforces the notion that the pathophysiology of EDs partially involves a process in which the perceptual amplification of certain physiological cues induces hyperreactivity and fearful responses. If such responses were generalized, individuals with EDs might be expected to show exaggerated interoceptive reactivity to changes not only in gut signals, but also to other interoceptive signals such as the breath and heartbeat, that contribute to emotional experience.

The results of this study point to a heightened sensitivity to dyspnea sensations at the self-report level in individuals with eating disorders, which may more broadly represent a difficulty adapting to internal shifts from homeostatic set points (as discussed in [68]). From this perspective, although speculative, we suspect that targeting heightened affective reactivity to the perturbation of interoceptive signals through interventions such as interoceptive exposure (as is being increasingly suggested [78, 79]) might help individuals with EDs to increase the perceived tolerance of discomforting body states. Whether such interventions might reduce pre-meal anxiety [80] and ultimately influence the avoidance of food consumption awaits empirical study.

### Limitations

Several limitations of this study warrant acknowledgments. First, individuals in the ED sample had comorbid mood and anxiety disorders according to the structured clinical interviews employed. Allowing comorbid depression into the samples might be seen as limiting the ability to determine whether heightened aversive experience of respiratory perturbation is related primarily to eating or anxiety-related pathology. However, this limitation is tempered by 1) the fact that the majority of individuals with eating disorders have mood related diagnoses when assessed under structured conditions [81, 82], 2) the fact that ED group was shown to have both heightened suffocation feelings (relative to the MA group), and 3) the fact that the ED group was the only group to show a correlation between suffocation fear and AS physical concerns. Second, the majority of individuals in this study were female, and thus further study may be need to determine whether these findings apply to males with ED. Third, although the lack of physiological differences in several of our physiological variables (including “heartrate change from baseline to minimum heartrate” and “change in pre/post O_2_/CO_2_ saturation”) could be limited by higher percentages of missing data (16–30% missing and 19–23% missing, respectively), our suffocation feeling/fear symptom-level measures had the lowest levels of missing data (<1%) for each variable. Lastly, the current study design is unable to distinguish whether heightened suffocation fear in ED is due to a premorbid process or whether it is a residual “scar” of the disorder [83]. Future research could address this limitation through longitudinal assessments of adolescents to see whether abnormal respiratory interoception precedes ED onset, or alternatively, evaluate the relationship between suffocation fear and clinical severity across periods of relapse, remission, and recovery [84].

## Conclusion

Individuals with ED and MA show evidence of heightened suffocation fear during inspiratory breath holding. Abnormal respiratory interoceptive processing may be an overlooked feature of ED psychopathology that warrants further investigation.

## Supporting information

S1 Fig AN subgroup analysis: Average feelings of suffocation and suffocation fear. Ratings Range from 0 (not at all)– 100 (extremely). HC = Healthy Comparison. MA = Mood/Anxiety. AN = Anorexia Nervosa. * indicates significant difference at p < 0.05.
(PDF)

S1 Table Anorexia nervosa subgroup analysis: Demographics and screening scores at study entry. HC = Healthy Comparison, MA = Mood/Anxiety, ED = Eating Disorder, BMI = Body Mass Index, SCOFF = Sick, Control, One Stone, Fat, Food Eating Disorder Screener, OASIS = Overall Anxiety Severity and Impairment Scale, PHQ-9 = Patient Health Questionnaire, ASI = Anxiety Sensitivity Index. ^a^ED>MA>HC, ^b^ED and MA > HC. Bolded values indicate significance at *p* < 0.05.
(PDF)

S2 Table AN symptom level subgroup analysis: t-tests to discern where group differences lie. HC = Healthy Comparison, MA = Mood/Anxiety. AN = Anorexia Nervosa. *d* = Cohen’s *d* effect size. 95% CI = 95% confidence interval for effect size. Symptom ratings for each individual were averaged over Breath Hold Trials 1 and 2. Bolded values indicate significance at *p* < 0.05.
(PDF)

S3 Table Spearman correlations between suffocation fear and anxiety sensitivity across groups. HC = Healthy Comparison. MA = Mood/Anxiety, ED = Eating Disorder. *p* = *p* value uncorrected. *p*_*corr*_ = *p* value with Benjamini-Hochberg False Discovery Rate Correction applied. Bolded values indicate significance at *p <* 0.05.
(PDF)

S4 Table Means and standard deviations (SD) for carbon dioxide percentage at baseline and pre/post carbon dioxide percent change for each breath hold trial. BH1 = Breath Hold Trial 1. BH2 = Breath Hold Trial 2. HC = Healthy Comparison. MA = Mood/Anxiety. ED = Eating Disorder.
(PDF)

S1 File Dataset. (CSV)

S2 File Variable names. contains an explanation of each variable name in the dataset S1 File.
(CSV)

S3 File Missing data. A table detailing missing data for each variable by participant group.
(CSV)
