# Supplementary figures and images for: Heightened affective response to perturbation of respiratory but not pain signals in eating, mood, and anxiety disorders
Source: PLoS One. 2020 Jul 15;15(7):e0235346. doi: 10.1371/journal.pone.0235346 (PMC7363095; doi:10.1371/journal.pone.0235346)

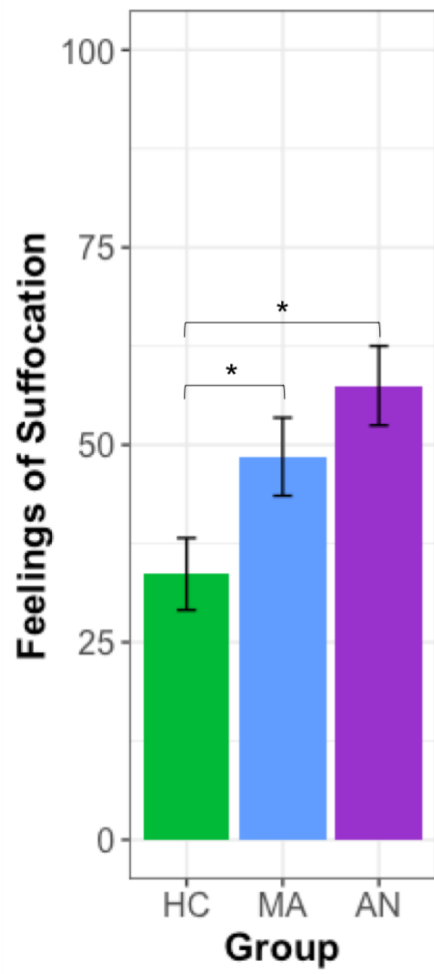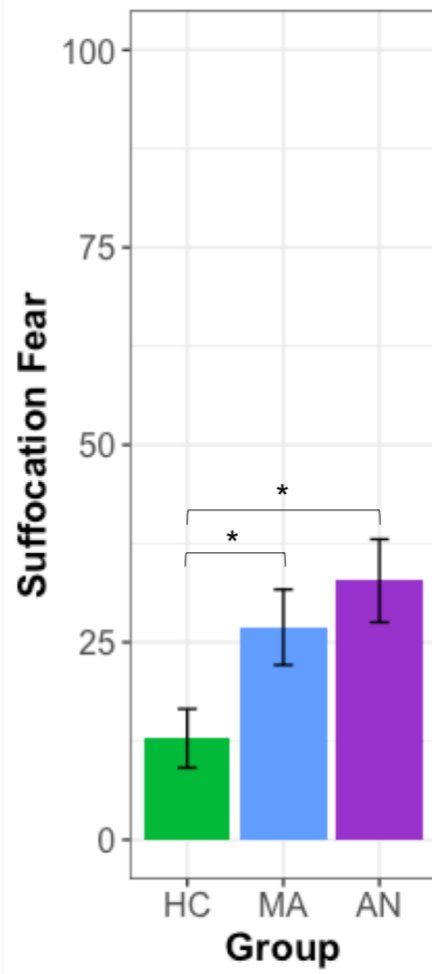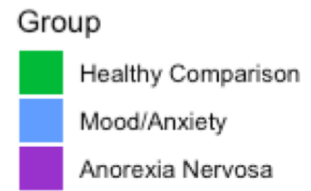

Supplement: S1 Fig — Ratings Range from 0 (not at all)– 100 (extremely). HC = Healthy Comparison. MA = Mood/Anxiety. AN = Anorexia Nervosa. * indicates significant difference at p < 0.05. (PDF) [file pone.0235346.s001.pdf]
