# Supplementary material for: Heightened affective response to perturbation of respiratory but not pain signals in eating, mood, and anxiety disorders
Source: PLoS One. 2020 Jul 15;15(7):e0235346. doi: 10.1371/journal.pone.0235346 (PMC7363095; doi:10.1371/journal.pone.0235346)
Supplement: S1 Table — HC = Healthy Comparison, MA = Mood/Anxiety, ED = Eating Disorder, BMI = Body Mass Index, SCOFF = Sick, Control, One Stone, Fat, Food Eating Disorder Screener, OASIS = Overall Anxiety Severity and Impairment Scale, PHQ-9 = Patient Health Questionnaire, ASI = Anxiety Sensitivity Index. aED>MA>HC, bED and MA > HC. Bolded values indicate significance at p < 0.05. (PDF) [file pone.0235346.s002.pdf]

S1 Table. Anorexia Nervosa Subgroup Analysis: Demographics and Screening Scores at Study Entry

	Mean (SD) HC	MA	AN	*F*	*df*	*p*
**Sample Size**	**(n = 30)**	**(n = 30)**	**(n = 30)**			
**Demographics**						
Age, years	26.27 (7.81)	27.67 (9.14)	25.90 (7.97)	0.38	2, 87	0.69
BMI	22.31 (3.50)	21.78 (3.10)	21.37 (3.32)	0.60	2, 87	0.55
**Clinical Measures**						
SCOFF[a]	0.13 (0.43)	0.73 (0.98)	2.77 (1.55)	48.41	2, 87	**<0.0001**
OASIS[b]	1.03 (1.49)	9.77 (3.19)	9.43 (3.88)	80.13	2, 87	**<0.0001**
PHQ[b]	0.67 (1.21)	12.53 (4.74)	10.27 (5.74)	62.78	2, 87	**<0.0001**
ASI-Total[b]	6.87 (5.20)	29.13 (15.45)	28.83 (15.19)	29.53	2, 86	**<0.0001**
ASI - Physical[b]	0.87 (1.04)	7.07 (5.65)	7.00 (6.76)	14.55	2, 86	**<0.0001**
ASI – Cognitive[b]	0.80 (1.63)	7.9 (7.20)	8.17 (5.50)	18.44	2, 86	**<0.0001**
ASI – Social[b]	5.20(3.61)	14.17 (6.12)	13.66 (4.78)	30.93	2, 86	**<0.0001**
**Diagnoses**		**n (%)**	**n (%)**			
Anorexia Nervosa	-	-	30 (100%)			
Major Depressive Disorder - Lifetime	-	27 (90%)	29 (97%)	-	-	-
Generalized Anxiety Disorder	-	13 (43%)	20 (67%)	-	-	-
Social Phobia	-	9 (30%)	7 (23%)	-	-	-
Panic Disorder	-	5 (17%)	1 (0.3%)	-	-	-
Agoraphobia	-	1 (0.3%)	0 (0%)	-	-	-
Post-Traumatic Stress Disorder	-	10 (33%)	9 (30%)	-	-	-
Alcohol/Substance Use Disorder Lifetime	-	3 (0.1%)	3 (0.1%)	-	-	-
Obsessive Compulsive Disorder	-	0 (0%)	1 (0.3%)	-	-	-

note: HC = Healthy Comparison, MA = Mood/Anxiety, ED = Eating Disorder, BMI = Body Mass Index, SCOFF = Sick, Control, One Stone, Fat, Food Eating Disorder Screener, OASIS = Overall Anxiety Severity and Impairment Scale, PHQ = Patient Health Questionnaire, ASI = Anxiety Sensitivity Index. [a]ED>MA>HC, [b]ED and MA > HC. Bolded values indicate $p < 0.5$.
