# Supplementary material for: Heightened affective response to perturbation of respiratory but not pain signals in eating, mood, and anxiety disorders
Source: PLoS One. 2020 Jul 15;15(7):e0235346. doi: 10.1371/journal.pone.0235346 (PMC7363095; doi:10.1371/journal.pone.0235346)
Supplement: S2 Table — HC = Healthy Comparison, MA = Mood/Anxiety. AN = Anorexia Nervosa. d = Cohen’s d effect size. 95% CI = 95% confidence interval for effect size. Symptom ratings for each individual were averaged over Breath Hold Trials 1 and 2. Bolded values indicate significance at p < 0.05. (PDF) [file pone.0235346.s003.pdf]

S2 Table. AN Symptom Level Subgroup Analysis: t-tests to Discern where Group Differences Lie

Task	Source	*t*	*DF*	*p*	*d* (95% CI)
Breath Hold	**Feelings of Suffocation: HC vs. MA**	-2.21	58	**0.03**	**0.56** (0.03 - 1.08)
	**Feelings of Suffocation: HC vs. AN**	-3.51	57	**0.001**	**0.91** (0.35 - 1.46)
	Feelings of Suffocation: MA vs. AN	-1.27	58	0.21	0.32 (-0.19 - 0.83)
	**Suffocation Fear: HC vs. MA**	-2.32	55	**0.02**	**0.60** (0.07 - 0.83)
	**Suffocation Fear: HC vs. AN**	-3.09	52	**0.003**	**0.80** (0.25 - 1.34)
	Suffocation Fear: MA vs. AN	-0.83	57	0.41	0.21 (-0.30 - 0.71)

note. HC = Healthy Comparison, MA = Mood/Anxiety. AN = Anorexia Nervosa. *d* = Cohen's *d* effect size. 95% CI = 95% confidence interval for effect size.Symptom ratings for each individual were averaged over Breath Hold Trials 1 and 2. See supplemental figure 2 for visual representation. Bolded values indicate $p < 0.5$.
