# Supplementary material for: Heightened affective response to perturbation of respiratory but not pain signals in eating, mood, and anxiety disorders
Source: PLoS One. 2020 Jul 15;15(7):e0235346. doi: 10.1371/journal.pone.0235346 (PMC7363095; doi:10.1371/journal.pone.0235346)
Supplement: S3 Table — HC = Healthy Comparison. MA = Mood/Anxiety, ED = Eating Disorder. p = p value uncorrected. pcorr = p value with Benjamini-Hochberg False Discovery Rate Correction applied. Bolded values indicate significance at p < 0.05. (PDF) [file pone.0235346.s004.pdf]

S3 Table. Spearman Correlations Between Suffocation Fear and Anxiety Sensitivity Across Groups

ASI-3 Domain	Group	r_s	p	p_{corr}
Total	HC	0.11	0.44	0.58
Cognitive	HC	0.06	0.66	0.79
Physical	HC	0.16	0.27	0.53
Social	HC	0.13	0.38	0.56
Total	MA	0.20	0.19	0.40
Cognitive	MA	0.20	0.17	0.40
Physical	MA	-0.03	0.85	0.90
Social	MA	0.02	0.89	0.90
Total	ED	0.34	0.02	0.06
Cognitive	ED	0.15	0.31	0.53
**Physical**	ED	0.50	**<0.0001**	**<0.0001**
Social	ED	0.34	0.02	0.06

HC = Healthy Comparison. MA = Mood/Anxiety, ED = Eating Disorder. p = p value uncorrected. p_{corr} = p value with Benjamini-Hochberg False Discovery Rate Correction applied. Bolded values indicate $p < 0.05$.
