# Supplementary material for: Heightened affective response to perturbation of respiratory but not pain signals in eating, mood, and anxiety disorders
Source: PLoS One. 2020 Jul 15;15(7):e0235346. doi: 10.1371/journal.pone.0235346 (PMC7363095; doi:10.1371/journal.pone.0235346)
Supplement: S4 Table — BH1 = Breath Hold Trial 1. BH2 = Breath Hold Trial 2. HC = Healthy Comparison. MA = Mood/Anxiety. ED = Eating Disorder. (PDF) [file pone.0235346.s005.pdf]

S4 Table. Means and Standard Deviations (SD) for Carbon Dioxide Percentage at Baseline and Pre/Post Carbon Dioxide Percent Change for Each Breath Hold Trial

Group	BH1 Baseline CO_2 Mean (SD)	BH2 Baseline CO_2 Mean (SD)	BH1 Delta CO_2 Mean (SD)	BH2 Delta CO_2 Mean (SD)
HC	4.40 (0.60)	4.40 (0.60)	1.50 (0.80)	1.40 (0.70)
MA	4.30 (0.70)	4.20 (0.50)	1.40 (0.60)	1.40 (0.60)
ED	4.30 (0.60)	4.40 (0.70)	1.10 (1.20)	1.20 (0.80)

BH1 = Breath Hold Trial 1. BH2 = Breath Hold Trial 2. HC = Healthy Comparison. MA = Mood/Anxiety. ED = Eating Disorder. Average percent exhaled CO_2 is 5 – 6%.
